# Comprehensive profiling analysis of the N6-methyladenosine-modified circular RNA transcriptome in cultured cells infected with Marek’s disease virus

**DOI:** 10.1038/s41598-021-90548-1

**Published:** 2021-05-26

**Authors:** Aijun Sun, Rui Wang, Shuaikang Yang, Xiaojing Zhu, Ying Liu, Man Teng, Luping Zheng, Jun Luo, Gaiping Zhang, Guoqing Zhuang

**Affiliations:** 1 College of Veterinary Medicine, Henan Agricultural University, Zhengzhou, 450002 Henan People’s Republic of China; 2 International Joint Research Center of National Animal Immunology, College of Veterinary Medicine, Henan Agricultural University, Zhengzhou, 450002 Henan People’s Republic of China; 3 Key Laboratory of Animal Immunology, Ministry of Agriculture and Rural Affairs and Henan Provincial Key La-boratory of Animal Immunology, Henan Academy of Agricultural Sciences, Zhengzhou, 450002 People’s Republic of China; 4 UK-China Centre of Excellence for Research on Avian Diseases, Henan Academy of Agricultural Sciences, Zhengzhou, 450002 People’s Republic of China; 5 College of Animal Science and Technology, Henan University of Science and Technology, Luoyang, 471003 People’s Republic of China

**Keywords:** Genetics, Microbiology, Molecular biology

## Abstract

Marek’s disease virus (MDV) induces severe immunosuppression and lymphomagenesis in the chicken, its natural host, and results in a condition that investigated the pathogenesis of MDV and have begun to focus on the expression profiling of circular RNAs (circRNAs). However, little is known about how the expression of circRNAs is referred to as Marek’s disease. Previous reports have is regulated during MDV replication. Here, we carried out a comprehensive profiling analysis of N6-methyladenosine (m^6^A) modification on the circRNA transcriptome in infected and uninfected chicken embryonic fibroblast (CEF) cells. Methylated RNA immunoprecipitation sequencing (MeRIP-Seq) revealed that m^6^A modification was highly conserved in circRNAs. Comparing to the uninfected group, the number of peaks and conserved motifs were not significantly different in cells that were infected with MDV, although reduced abundance of circRNA m^6^A modifications. However, gene ontology and Kyoto encyclopedia of genes and genomes (KEGG) pathway analyses revealed that the insulin signaling pathway was associated with the regulation of m^6^A modified circRNAs in MDV infection. This is the first report to describe alterations in the transcriptome-wide profiling of m^6^A modified circRNAs in MDV-infected CEF cells.

## Introduction

Marek’s disease virus (MDV) causes a fatal disease that is referred to as Marek’s disease (MD). This is a disease that is characterized by immunosuppression, neurological disorders, and T-cell lymphoma formation^1–3^. The genome of MDV is approximately 180 kb in size and includes a unique long region (UL) and a unique short region (US), with reverse repetitive complementary sequences at both ends of the genome, and encoding more than 100 genes^4,5^. MDV can be classified as serotype 1 (MDV-1), serotype 2 (MDV-2), and serotype 3 (MDV-3). MDV-2 and MDV-3 are non-pathogenic while MDV-1 causes a diverse degree of diseases and tumors once it has infected its natural host^6^. Although progress has been made in terms of analyzing gene function in MDV, the precise mechanisms involved in the pathogenesis of this disease have yet to be determined.

Non-coding RNAs (ncRNAs), including microRNAs (miRNAs), long non-coding RNAs (lncRNAs), and circular RNAs (circRNAs), are known to play important regu-latory roles in a range of diseases^7^. CircRNAs are a class of long RNA molecules that are over 200 nucleotides in length and are widely expressed in eukaryotes with a closed ring structure, lacking a 5′-cap structure, and a 3′-poly(A) tail. CircRNAs are formed by exon, intron, and exon–intron sequences, and are mainly located in the cytoplasm for post-transcriptional regulation; in this region, circRNAs are stable and not easily degraded by RNA exonuclease^8^. Some circRNAs have been found in cell nuclei, thus supporting a potential role in the regulation of transcription^9^. It has been reported that circRNA acts as a sponge to regulate the expression of miRNAs^10^. Interestingly, virus infection has been shown to alter the landscape of the circRNAs transcriptome may help the virus to escape immune surveillance. It has been demonstrated that circRNAs are also involved in ALV-J-induced tumorigenesis in susceptible and resistant chickens^11^. In MD-induced spleen tumors, a total of 2169 circRNAs have been identified; these were derived from exons. When comparing spleen samples from survivors and non-infected chickens, 113 circRNAs were identified to be abnormally expressed. Comprehensive analysis revealed that circRNAs may also participate in tumorigenesis by regulating the regulatory network associated with the immune response^12,13^. However, we do not yet know how circRNAs are regulated during MDV infection.

Recent studies have identified that RNA modifications can regulate the epigenetic To date, at least 100 RNA modifications have been reported in biological and pathological activities^14^. In eukaryotes, 5′-Cap and 3′-poly(A) tail modifications can play an important role in the regulation of transcription, while the internal modification of messenger RNA (mRNA) is used to maintain stability. The most common internal modifications of mRNA include N6-methyladenosine (m^6^A), N1-methyladenosine (m^1^A), and N5-methylcytosine (m^5^C)^15^. m^6^A is the most common reversible base modification on RNA and can affect transcription, splicing, localization, translation, structure stability, and the post-transcriptional regulation of gene expression. m^6^A modification has also been detected on ncRNAs, such as transfer RNA (tRNA) and ribosomal RNA (rRNA)^16^. Many enzymes have been identified in m^6^A modification, including methyltransferase and demethylase. Methyl-transferase, which acts as a writer, is an important catalytic enzyme complex that causes m^6^A methylation of bases on RNA, including components of METTL3, METTL14, WTAP, and KIAA1492, and other unknown proteins^17^. The m^6^A demethylase complex contains FTO and ALKBH5 proteins and is referred to as an eraser. FTO was first characterized as a member of the Alkb protein family and was associated with obesity. Subsequently, FTO was confirmed as a very important component of the demethylase complex^18–20^. In addition, m^6^A-modified mRNA needs a specific RNA binding protein-methylated reading protein, which is known as a reader. A variety of reading proteins have been identified by RNA pull-down assays, including YTH domain protein, nuclear heterogeneous ribosomal protein (hnRNP), and eukaryotic initiation factor (EIF). These reading proteins can specifically bind to the m^6^A methylation region, thus weakening homologous binding to RNA-binding proteins and changing the secondary structure of the RNA to regulate interactions between protein and RNA^15,21^.

Previous studies of m^6^A modification mainly focused on the maintenance of mRNA stability, mRNA precursor splicing, polyadenylate acidification, mRNA transport, and the initiation of translation. In addition, research has shown that ncRNAs also exhibit a large number of base modification activities after transcription^22^. It has been shown that abnormal patterns of m^6^A modification are involved in a series of diseases, including tumors and viral infections^23^. In this study, we investigated the expression profile of circRNA and comprehensively analyzed the m^6^A modification of circRNAs in MDV-infected CEF cells. We also analyzed the signaling pathways associated with the m^6^A modification of circRNAs and the roles of m^6^A modification in MDV infection.

## Materials and methods

### Cells and virus

Specific-pathogen free (SPF) eggs were purchased from Boehringer Ingelheim (Beijing, China) and incubated at 37 °C in order to isolate primary chicken embryo fibroblast (CEF) cells^24^, as described previously. CEF cells were prepared after 9 days of incubation, and were then cultured in Dulbecco’s modified essential medium (DMEM) (Solarbio, Beijing, China) containing 5% fetal bovine serum (FBS) (Gibco, CA, USA), 100 U/mL of penicillin and 100 µg/mL of streptomycin at 37 °C with 5% CO_2_. Secondary CEF cells were seeded to 80–90% confluency in 75 cm^2^ petri dishes. The cells were then inoculated with 10^6^ plaque formation units (PFU) of Md5 (Passage two), Uninfected CEF cells were used as negative control. Seven days post-infection, CEF cells were harvested when cytopathic effects (CPE) became clearly visible in approximately 70–80% of Md5-infected cells.

### RNA isolation

Total RNA was extracted from CEF cells using TRIzol reagent (Invitrogen, Carlsbad, CA, USA). The RNA concentration of each sample was then determined with a NanoDrop ND-1000 (Thermo Fisher Scientific, MA, USA). The quality of the RNAs was subsequently identified by measuring the OD260/OD280 value, and RNA purity was confirmed by measuring the OD260/OD230 value. RNA integrity and gnomic DNA contamination were measured by denatured agarose gel electrophoresis.

### Methylated RNA immunoprecipitation sequencing (MeRIP-Seq)

Fragmented RNA was incubated with an anti-m^6^A polyclonal antibody (Synaptic Systems, 202003) in immunoprecipitation (IPP) buffer for 2 h at 4 °C^25^. The reaction mixture was then immunoprecipitated with protein A magnetic beads (Thermo Fisher, MA, USA) at 4 °C for 2 h. Next, the bound RNA was eluted from the beads with N6-methyladenosine antibody in IPP buffer and extracted with TRIzol reagent (Thermo Fisher, MA, USA). The extracted RNA was then prepared with a NEBNext Ultra II Directional RNA Library Prep Kit (NEB, MA, USA). Both the input sample (without immunoprecipitation) and the m^6^A immunoprecipitational samples were subjected to 150 bp paired-end sequencing on an Illumina HiSeq sequencer.

### Data analysis

Paired-end reads were acquired from an Illumina HiSeq 4000 sequencer and were quality controlled by Q30. Next, 3′ adaptor-trimming and low quality reads were removed by cutadapt software (v1.9.3, https://github.com/marcelm/cutadapt/) to obtain high quality clean reads. The reads were then aligned to the reference genome (Gal5; GCA_000002315.3) with Hisat2 software (v2.0.4). Expressed circRNAs were identified using input reads. Methylated sites on the circRNA were then identified by the MeTPeak package in the R environment. The MeTDiff package was used to investigate the differential methylation of circRNAs. The Gene Ontology (GO) (http://www.geneontology.org) and the Kyoto Encyclopedia of Genes and Genomes (KEGG) (https://david.ncifcrf.gov) pathway enrichment analyses were performed to identify differentially methylated genes^26^. The interactive analysis tool Integrative Genomics Viewer (IGV) (v2.4.10, http://www.igv.org/) software was used to visualize the alignments on the genome.

## Results

### Transcriptome-wide analysis of circRNAs in Md5-infected CEF cells

**Figure 1:**
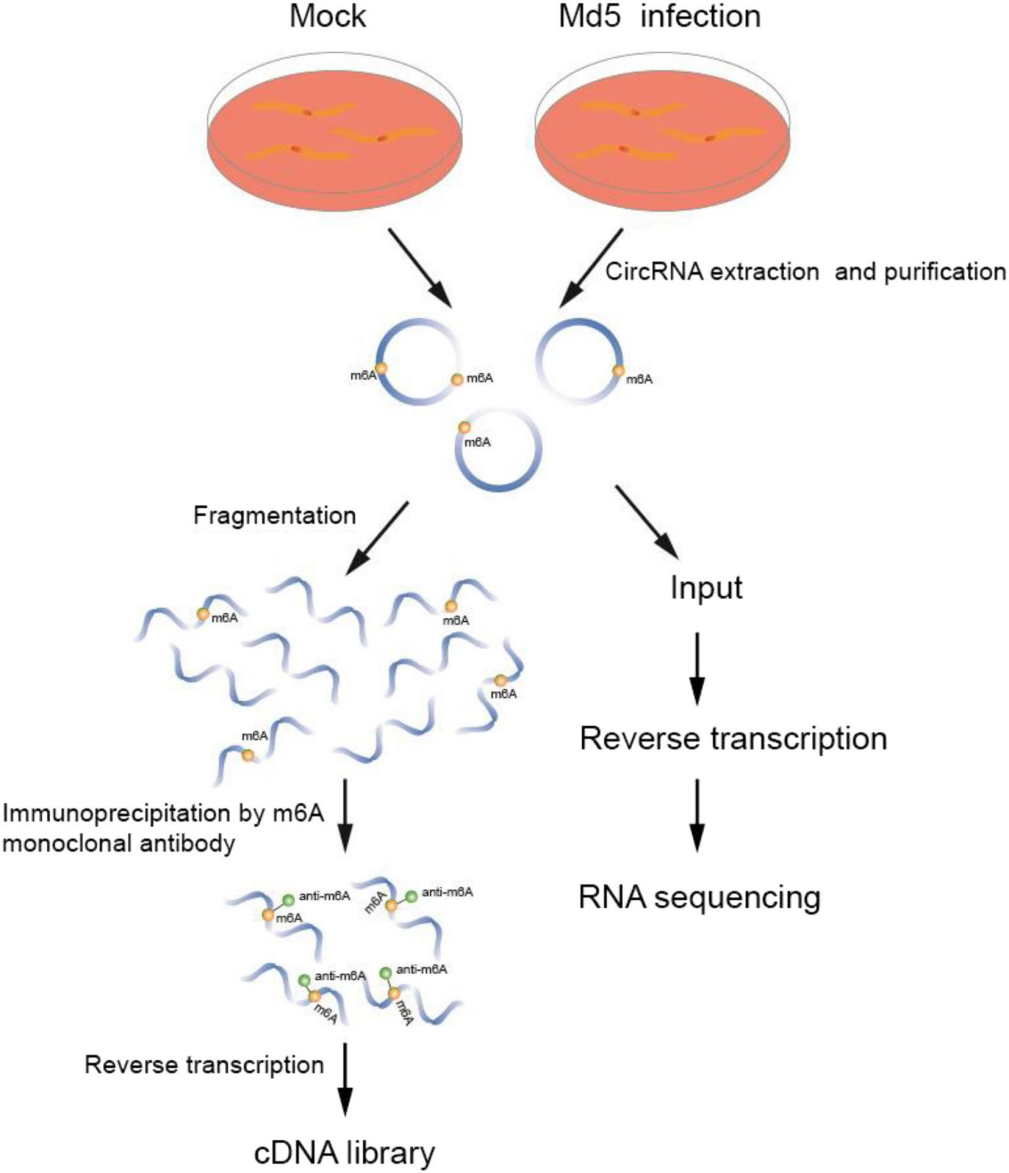
Flowchart depicting construction of cDNA libraries used for m^6^A-modified circRNA transcriptome sequencing of uninfected and Md5-infected CEF cells. (Figure was made by Adobe Illustrator, v 24.0, https://www.adobe.com/cn/products/illustrator.html).

**Figure 2:**
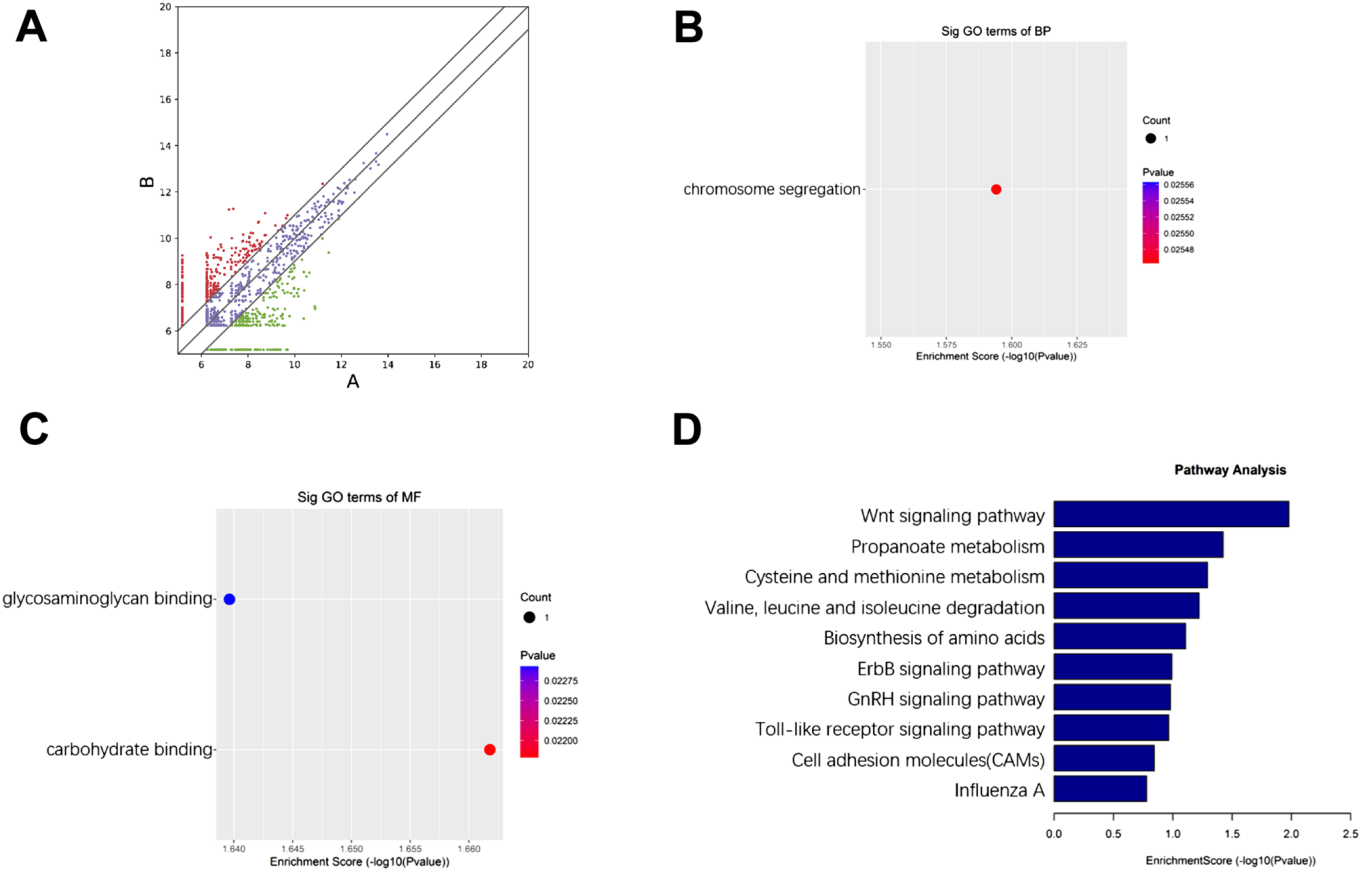
(**A**) Differentially expressed circRNAs in infected and control groups. The X axis represents the expression level of the gene in the control group, and the Y axis represents the expression level of the gene in the infected group. A represents the uninfected group and B represents the infected group. Red dots represent up-regulated genes in group B compared with group A. Green dots represent down-regulated genes in group B compared with group A. Blue dots represent genes with no significant differences in group B compared with group A. (Made by edgeR, v3.16.5, http://www.bioconductor.org/packages/release/bioc/html/edgeR.html) (**B**,**C**) GO enrichment of host genes associated with down-regulated circRNAs for (**B**) biological processes and (**C**) molecular functions; (**D**) KEGG pathway analysis of the host genes for circRNAs.

For circRNA transcriptome profiling analysis, we first extracted RNA from the MDV infected group and control group. After RNA fragmentation, RNA were seperatee to two parts. One was used for regular circRNA transcriptome analysis as input. The other part was enriched by MeRIP-seq assay for m^6^A modified circRNA transcriptome analysis (Fig. 1). A total of 6045 circRNAs, with at least two independent reads, were identified, ranging from 131 nucleotides to 96,080 nucleotides. The circRNAs were derived from exonic, intronic, intergenic, sense overlapping, and antisense regions (Fig. 2A). GO enrichment analysis for biological processes (BP) indicated that genes related to chromosome segregation were up-regulated (Fig. 2B). For molecular functions (MF), genes related to carbohydrate binding were up-regulated, while genes related to glycosaminoglycan binding were down-regulated (Fig. 2C). For cellular components (CC), no up-regulated or down-regulated genes related to the relevant signaling pathway were detected. We performed KEGG enrichment analysis for the differentially expressed circRNAs, and identified a range of affected pathways, including the Wnt signaling pathway and propanoate metabolism; along with the degradation and metabolism of cysteine, methionine, valine, leucine, and isoleucine. We also identified changes in the biosynthesis of amino acids, the ErbB signaling pathway, the GnRH signaling pathway, the Toll-like receptor signaling pathway, cell adhesion molecules (CAMs), influenza A, and the MAPK signaling pathway (Fig. 2D).

### Epitranscriptome-wide m^6^A modification analysis of circRNAs in Md5-infected CEF cells

**Figure 3:**
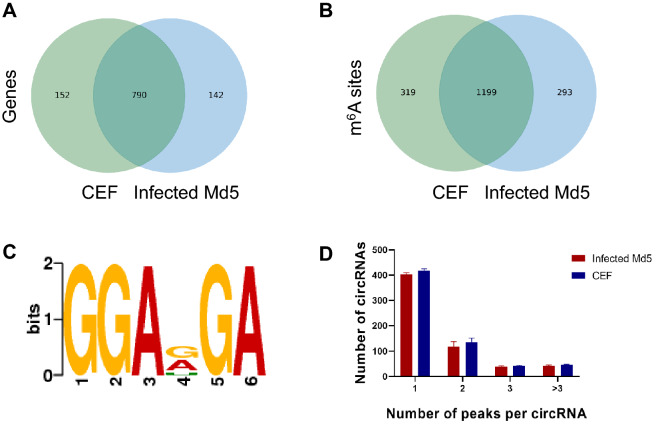
The characteristics of m^6^A peaks. (**A**) Venn diagram of m^6^A genes in infected Md5 and CEF groups; (**B**) Venn diagram of m^6^A methylation sites identified in circRNAs from infected and CEF groups; (**C**) The sequence motif of m6A sites in infected and control groups (Made by Discriminative Regular Expression Motif Elicitation (DREME), v5.3.3, https://meme-suite.org/meme/tools/dreme); (**D**) Proportion of genes harboring different numbers of m^6^A sites in the two groups (Made by Prism, v8.0.2, https://www.graphpad.com).

To investigate the potential regulatory role of m^6^A on circRNAs, we carried out transcriptome-wide m^6^A modification profiling analysis. We detected 790 genes from a total of 932 annotated genes (the infected group) and 942 annotated genes (from the control group) in both the uninfected and Md5-infected CEF cells groups (Fig. 3A). We also detected 1199 from a total of 1483 and 1509 m^6^A modified peaks were in both the infected and control groups (Fig. 3B). m^6^A sites with a fold change (FC) > 2 were considered to be specific. Comparison of the infected group with control group revealed that 13% of the confirmed sites were specific to the infected group while 14% were specific to the control group. These results indicated that Md5 infection induced the reduction of the overall incidence of m^6^A modification in CEF cells.

To determine whether the m^6^A peak of circRNA featured a conserved RRACH sequence (in which the R represents purine, A represents m^6^A, and H is a non-guanine base). The sequences of the first 1000 peaks (50 bp on both sides of the peaks) with the largest enrichment factor in each group of samples were then selected and the sequences of these peaks were analyzed to identify meaningful motif sequences. We scanned part of the m^6^A modified peak sequence of the circRNA to determine whether the identified m^6^A peak contained the RRACH conservative motif sequence (where R represents purine, A represents m^6^A and H represents non-guanine bases). We found that GGAD (A, G, and U) GA was a reliable m^6^A modified motif in both the uninfected and Md5-infected groups obtained based on E-value (Fig. 3C).

The m^6^A peak abundance of circRNA was further determined, the 67.1% of circRNAs in the infected group contained one m^6^A peak, slightly higher than the 65.0% of circRNAs in the control group. The numbers of two peaks (19.7% in the infected group vs. 21.2% in the control group), three peaks (6.3% in the infected group vs. 6.5% in the control group), and more than three peaks (6.9% in the infected group vs. 7.3% in the control group) were also determined (Fig. 3D). and the MAPK signaling pathway (Fig. 2D).

### Heatmap of m^6^A levels of circRNAs in control and infected groups

**Figure 4:**
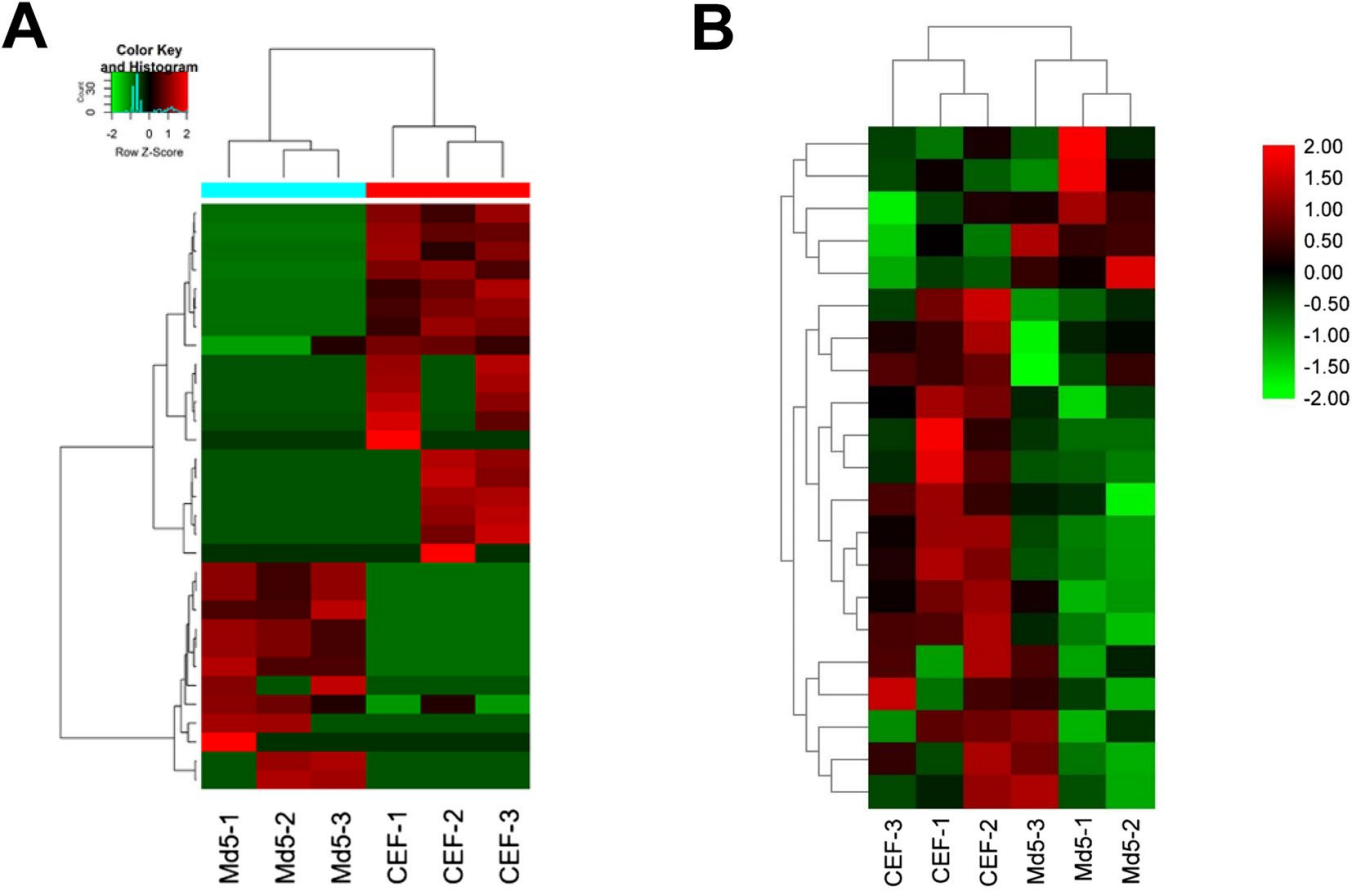
m^6^A modification clustering analysis. (**A**) Cluster analysis of m^6^A methylation at the transcriptome level. (**B**) Cluster analysis of m^6^A m6A modified lncRNA genes in the infected and control groups. The color represents the size of the log-fold enrichment (FE) value: the closer the color is to red, the larger the logFE value. Md5-1, Md5-2, Md5-3 represent the Md5-infected CEF group with three independent replicates. ( Made by Tbtools, v1.082, http://www.tbtools.org).

Based on unsupervised hierarchical cluster analysis, there were significant differences in expression when compared between the infected and control groups (Fig. 4A). At the transcriptomic level, a total of 31 differentially expressed circRNAs were detected, among which 12 were up-regulated and 19 were down-regulated. Heatmaps of m^6^A methylation levels showed differences in expression between the infected and control groups (Fig. 4B). Of the 56 differentially expressed methylation modifications, 21 m^6^A modified peaks were detected in the up-regulated genes (Table 1) and 35 m^6^A modified peaks were detected in the down-regulated genes (Table 2). These results indicated that the different clustering could clearly distinguish the level of transcriptome-wide m^6^A modification between the Md5-infected and uninfected groups.

**Table 1:** Top ten up-methylated peaks of differentially m^6^A methylated sites in the infected group.

chrom	txStart	txEnd	GeneName	Foldchange
1	32604598	32604680	–	128.7469112
1	91293301	9129320	ENSGALG00000024077	99.7
4	51575081	51575380	EPHA5	95.3
8	20814821	20815160	ARMH1	66.4
2	74540441	74540980	CDH18	60.7
5	42254121	42254360	FLRT2	14.30666667
2	1.07E+08	106671480	ENSGALG00000029474	12.02777778
1	1.77E+08	177137880	ENSGALG00000042343	9.117117117
2	2374581	2374900	GUK1	8.391304348
2	–	97042640	–	6.788732394

“–” represents not available**.** Chrom: Chromosome; TxStart/TxEnd: Start/end position of the differentially methylated RNA peaks.

**Table 2:** Top ten down-methylated peaks of differentially m^6^A methylated sites in the infected group.

chrom	txStart	txEnd	GeneName	Foldchange
1	4885621	4885840	PPARG	92.5
3	74518201	74518520	CDH18	71.9
19	83424141	83424380	CD200R1	65.7
21	19985261	19985480	PTPRF	22.3125
Z	8733401	8733640	ARPP19	20.34210526
13	30213841	30214120	IL15	10.22463768
2	16635161	16635420	ENSGALG00000029896	8.838095238
12	33409821	33410100	ENSGALG00000034760	7.806451613
2	24695481	24695720	SLC1A7	5.179153094
Z	11273981	11274180	–	5.04494382

“–” represents not available**.** Chrom: Chromosome; TxStart/TxEnd: Start/end position of the differentially methylated RNA peaks.

### Chromosome visualization of m^6^A in circRNA

**Figure 5:**
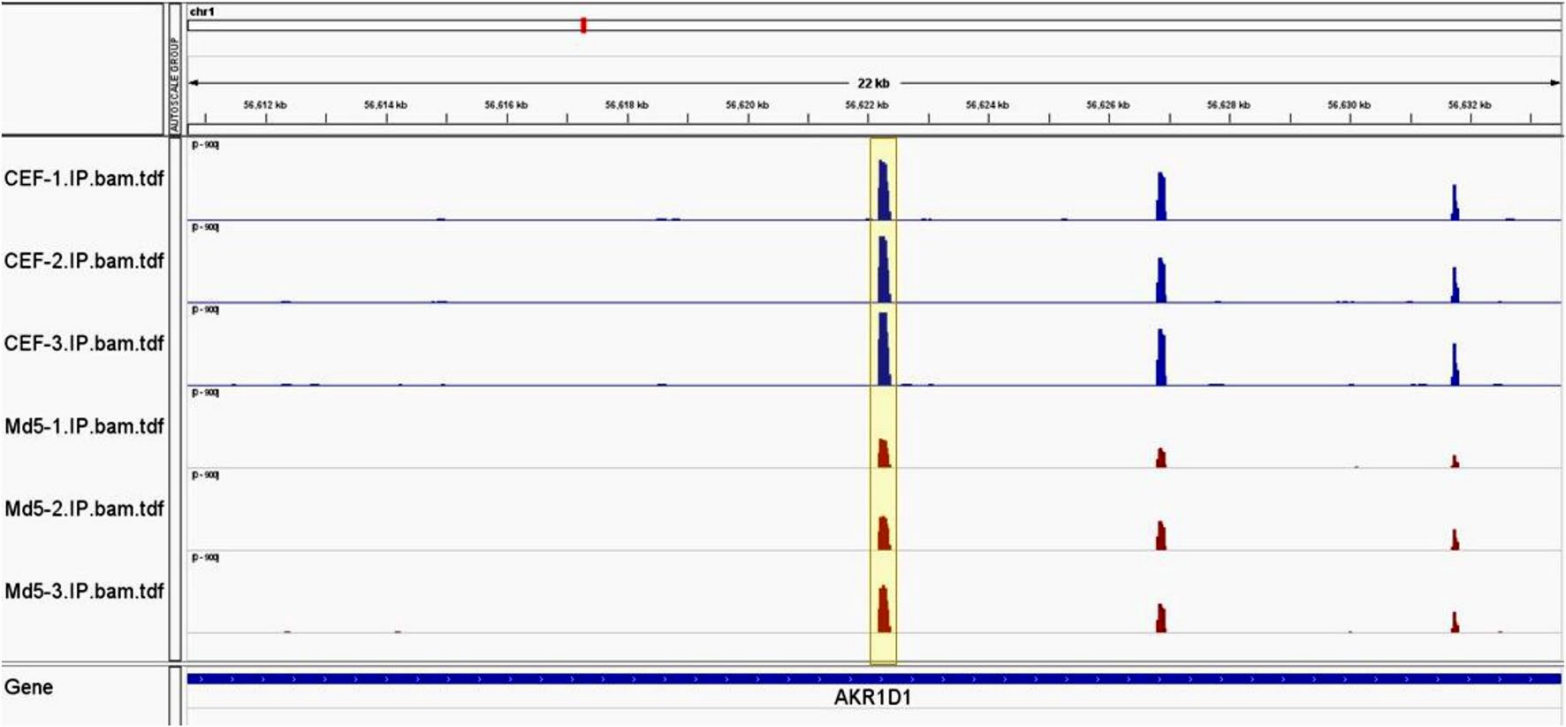
Chromosome visualization of m^6^A sites in lncRNAs. Red represents the infected group while blue represents the control group. Md5-1, Md5-2, Md5-3 represent the Md5-infected CEF group with three independent replicates. Differentially methylated m^6^A peaks visualized in Md5-infected group and control group. The highlighted area represents one of the differential methylation peaks.

To investigate the distribution of m^6^A methylation sites within the entire genome, we scanned the m^6^A modified sites on all chromosomes. As an example, the methylation level and distribution of m^6^A on AKR1D1 gene differed when compared between the infected and control groups (Fig. 5). These results indicated the differential functions of m^6^A modification when compared between these two groups.

### GO enrichment analysis of m^6^A in circRNAs

**Figure 6:**
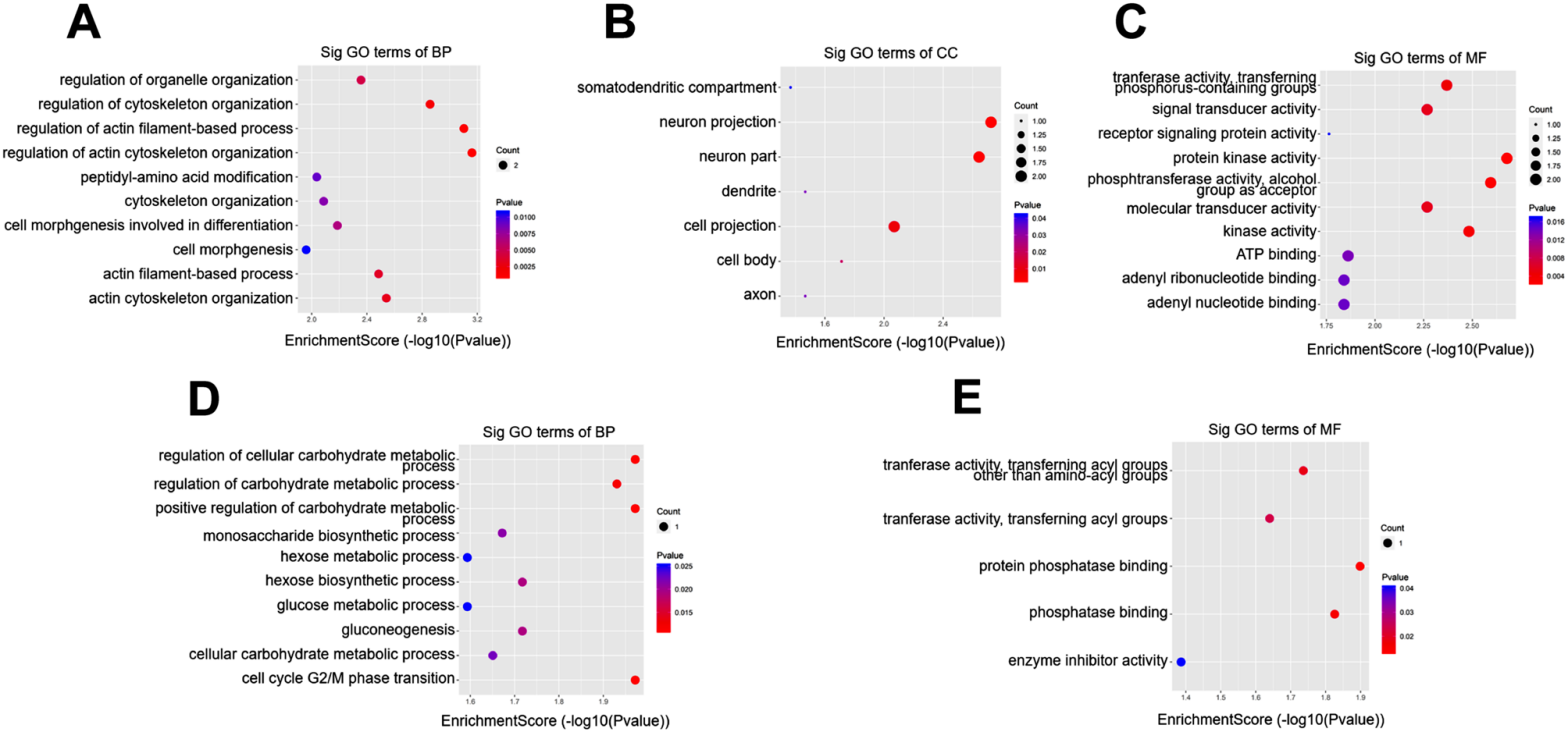
Gene ontology analyses of the infected and control groups. (**A**) The top ten gene ontology terms of biological processes were significantly enriched for the up-regulated genes; (**B**) The top seven gene ontology terms of cell component were significantly enriched for the up-regulated genes; (**C**) The top ten gene ontology terms of molecular functions were significantly enriched for the up-regulated genes; (**D**) The top ten gene ontology terms of biological process significantly enriched for down-regulated genes; (**E**) The top five gene ontology terms of molecular functions significantly enriched for down-regulated genes.

To examine the potential function of m^6^A modification in infected and control groups in vitro, we performed GO enrichment analysis. For BP, genes with up-regulated m^6^A sites were significantly enriched in organelle organization, cytoskeleton organization, actin filament-based process, actin cytoskeleton organization, peptidyl-amino acid modification, cytoskeleton organization, cell morphogenesis involved in differentiation, cell morphogenesis, actin filament-based process, and actin cytoskeleton organization (Fig. 6A). For cellular components (CC), genes with up-methylated m^6^A sites were mainly enriched in somatodendritic compartments, neuron projection, neuron part, dendrite cell projection, cell body, and axon; we did not detect the function of the methylated genes that were down-regulated (Fig. 6B). For MF, genes with up-regulated m^6^A sites were associated with transferase activity, transferring phosphorus-containing groups, signal transducer activity, receptor signaling protein activity, protein kinase activity, phosphotransferase activity, alcohol group as acceptor, molecular transducer activity, kinase activity, ATP binding, adenyl ribonucleotide binding and adenyl nucleotide binding (Fig. 6C). With regards to BP and down-regulated genes, m^6^A sites were highly enriched in cellular carbohydrate metabolic process, regulation of carbohydrate metabolic process, positive regulation of carbohydrate metabolic process, monosaccharide biosynthetic process, hexose metabolic process, hexose biosynthetic process, glucose metabolic process, gluconeogenesis, cellular carbohydrate metabolic process and cell cycle G2/M phase transition (Fig. 6D). With regards to MF and downregulated genes, m^6^A sites were enriched in transferase activity, transferring acyl groups other than aminoacyl groups, transferase activity, transferring acyl groups, protein phosphatase binding, phosphatase binding, and enzyme inhibitor activity (Fig. 6E).

### KEGG pathway analysis of m^6^A in circRNAs

**Figure 7:**
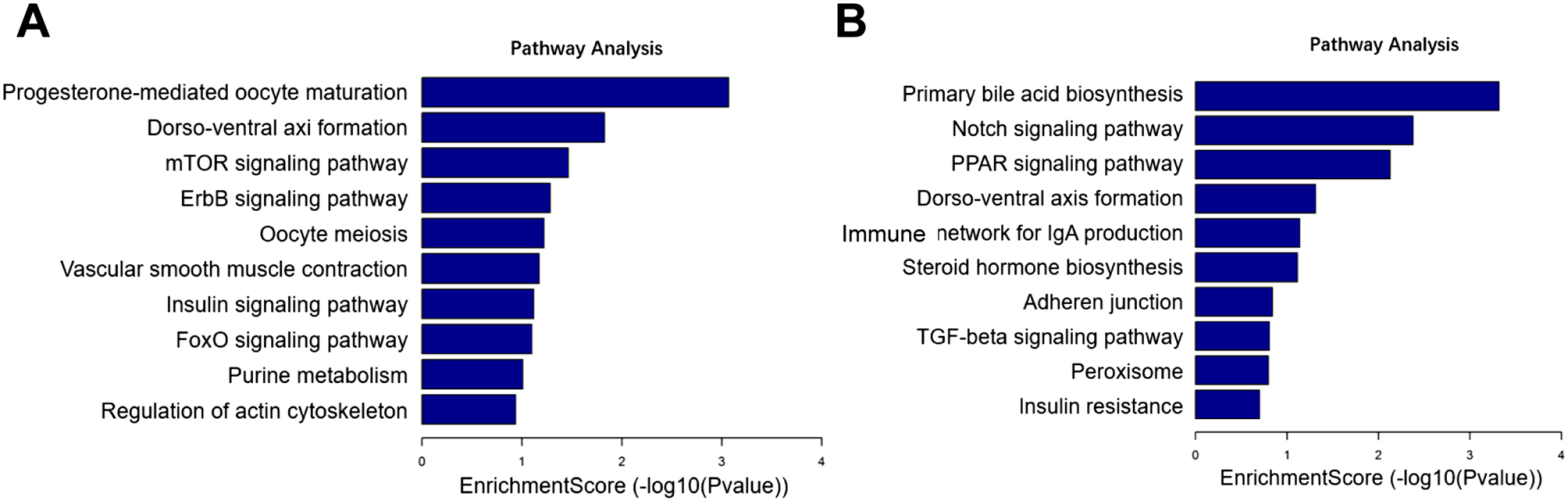
KEGG pathway analysis of differentially methylated m^6^A genes in circRNAs. (**A**) Bar plot showing the top 10 enrichment scores of significantly enriched pathways for up-methylated m^6^A genes in the infected group; (**B**) Bar plot showing the top 10 enrichment scores of significantly enriched pathways for down-methylated m^6^A genes in the infected group.

The presence of m^6^A is critical to the normal function of cells. For each differentially expressed m^6^A modification site, we investigated the correlation with each up-regulated or down-regulated circRNA. To further explore the function of m^6^A in infected and control groups, we performed KEGG enrichment analysis of differentially m^6^A modified genes. The top 10 pathways included progesterone-mediated oocyte maturation, dorsoventral axis formation, mTOR signaling pathway, ErbB signaling pathway, oocyte meiosis, vascular smooth muscle contraction, insulin signaling path-way, FoxO signaling pathway, purine metabolism, and regulation of actin cytoskeleton (Fig. 7A). The top 10 down-regulated pathways included primary bile acid biosynthesis, notch signaling pathway, PPAR signaling pathway, dorsoventral axis formation, immune network for IgA production, steroid hormone biosynthesis, adherens junction, TGF-beta signaling pathway, peroxisome and insulin resistance (Fig. 7B).

## Discussion

Recent research identified 2169 circRNAs were discovered in the comprehensive analysis of differentially expression profiling of the circRNA landscape in MDV-induced chicken tumorous spleens versus uninfected spleens^12^. In the present study, we identified circRNAs that were differentially expressed in MDV-infected CEF cells. We found most of the circRNAs were produced from exonic, intronic, intergenic, sense overlapping, and antisense regions. Previous research has revealed that differentially expressed circRNAs are enriched in the apoptotic processes, nucleic acid binding region, DNA repair, and immune response during tumorigenesis^12^. In MDV-infected CEF cells, however, genes related to chromosome segregation, carbohydrate binding, and glycosaminoglycan binding, were regulated. The differential expression profiling and function of circRNAs may be tissue- or tumorigenesis-specific. Furthermore, we investigated the differential expression profiling on m^6^A modified circRNAs. By measuring the degree of methylation in control and infected groups, we identified more than 1000 methylated peaks in circRNAs. We compared the sequences of the top ten peaks with the highest enrichment ratio of circRNA (50 bp on each side of the vertex). It was found that the GGAD (A, G, and U) GA sequence is one of the conserved motif sequences of circRNA based on E-value (Fig. 3C), which is different with canonical DRACH/RRACH consensus motif sequence of m^6^A deposition. Whether it is circRNA-specific motif sequence need to be further characterized.

Our results showed that the frequency of m^6^A methylation and the number of methylated genes were slightly lower in the infected group than in the control group. However, our clustering analysis identified significant differences in the methylation levels of individual genes between the infected and control groups. Meanwhile, KEGG analysis identified the differential expression of circRNAs that were associated with specific signaling pathways in MDV-infected CEF cells. Interestingly, the ErbB signaling pathway induced by MDV infection was also regulated by m^6^A modification. These data indicated the specific role of m^6^A modified circRNAs in the replication of MDV in cultured CEF cells. In addition, the insulin signaling pathway was up-regulated while the insulin resistance signaling pathway was down-regulated. Previous research has demonstrated that insulin-like growth factor is abundantly expressed in MDV-mediated immune suppression and vaccine responses^27^. Our results indicated that m^6^A modification regulated the insulin signaling pathway and that this might be important in MDV infection and immune evasion. However, the specific mechanism by which m^6^A modification acts on circRNA-associated signaling pathways has yet to be elucidated.

CircRNA can act as a miRNA sponge to affect gene expression in various biological and pathological activities^28–30^. CircRNAs can bind with miR-155 in MDV-induced tumorous spleens; this is an orthologue of MDV-encoded MDV-miR-M4^12,31^. Research has also shown that MDV-miR-M4 plays significant roles in tumorigenesis^32,33^. In MDV-infected CEF cells, however, we did not identify a correlation between circRNAs and miRNAs, thus indicating that the m^6^A modification on circRNAs may change the regulatory role on miRNAs^34,35^. It has also been demonstrated that miRNA can also be regulated by m^6^A modification. Whether m^6^A modified miRNA can affect gene expression in MDV infection needs to be further investigated.

It is of great important that we observed significantly higher expressions of METTL14 and ALBHK5 in MDV infected CEF cells comparing to uninfected control (Data not shown). These data suggest that MDV might change m^6^A modification of circRNAs through regulating activities of methyltransferase and demethylase to facilitate its replication and even pathogenesis. It is critical to know the detailed molecular mechanism of how MDV affect and regulate the circRNAs m^6^A modification in the next investigation. Meanwhile, the role of m^6^A modified circRNAs on MDV pathogenesis and even tumoregenesis also need to be further investigated.

## Conclusions

We used MeRIP-sequencing to analyze circRNA m^6^A modifications in Md5-infected and uninfected control groups. We then compared the differences in m^6^A modification between the two groups. We identified the relative abundances of m^6^A modification and conserved motifs in MDV-infected and control groups. GO and KEGG analysis showed that up-regulated and down-regulated methylation genes were mainly associated with virus infection. However, the detailed regulatory role of m^6^A modified circRNAs in MDV infection needs to be investigated further.

## Data Availability

All data generated or analyzed during this study are included in this submitted manuscript. The datasets generated and/or analyzed during the current study are available in the NCBI repository (https://www.ncbi.nlm.nih.gov/geo/). The data is accessible via NCBI GEO submission ID: GSE166240. To review GEO accession GSE166240: Go to https://www.ncbi.nlm.nih.gov/geo/query/acc.cgi?acc=GSE166240. Enter token klufyeaednulxgb into the box.
